# Transcriptome survey of Patagonian southern beech *Nothofagus nervosa* (= *N. Alpina*): assembly, annotation and molecular marker discovery

**DOI:** 10.1186/1471-2164-13-291

**Published:** 2012-07-02

**Authors:** Susana L Torales, Máximo Rivarola, María F Pomponio, Paula Fernández, Cintia V Acuña, Paula Marchelli, Sergio Gonzalez, María M Azpilicueta, Horacio Esteban Hopp, Leonardo A Gallo, Norma B Paniego, Susana N Marcucci Poltri

**Affiliations:** 1 Instituto de RecursosBiológicos, IRB, Instituto Nacional de Tecnología Agropecuaria (INTA Castelar), CC 25, Castelar, B1712WAA, Argentina; 2 Instituto de Biotecnología, CICVyA, Instituto Nacional de Tecnología Agropecuaria (INTA Castelar), CC 25, Castelar, B1712WAA, Argentina; 3 EEA Bariloche, Genética Ecológica y Mejoramiento Forestal, Instituto Nacional de Tecnología Agropecuaria (INTA, Bariloche), CC 277, 8400, Bariloche, Argentina; 4 Facultad de Ciencias Exactas y Naturales, Universidad de Buenos Aires, Buenos Aires, Argentina; 5 CONICET, Buenos Aires, Argentina

**Keywords:** Nothofagaceae, Forest genomics, Pyrosequencing, de novo transcriptome assembly, SSRs, Functional annotation

## Abstract

**Background:**

*Nothofagus nervosa* is one of the most emblematic native tree species of Patagonian temperate forests. Here, the shotgun RNA-sequencing (RNA-Seq) of the transcriptome of *N. nervosa*, including *de novo* assembly, functional annotation, and *in silico* discovery of potential molecular markers to support population and associations genetic studies, are described.

**Results:**

Pyrosequencing of a young leaf cDNA library generated a total of 111,814 high quality reads, with an average length of 447 bp. *De novo* assembly using Newbler resulted into 3,005 tentative isotigs (including alternative transcripts). The non-assembled sequences (singletons) were clustered with CD-HIT-454 to identify natural and artificial duplicates from pyrosequencing reads, leading to 21,881 unique singletons. 15,497 out of 24,886 non-redundant sequences or unigenes, were successfully annotated against a plant protein database. A substantial number of simple sequence repeat markers (SSRs) were discovered in the assembled and annotated sequences. More than 40% of the SSR sequences were inside ORF sequences. To confirm the validity of these predicted markers, a subset of 73 SSRs selected through functional annotation evidences were successfully amplified from six seedlings DNA samples, being 14 polymorphic.

**Conclusions:**

This paper is the first report that shows a highly precise representation of the mRNAs diversity present in young leaves of a native South American tree, *N. nervosa*, as well as its *in silico* deduced putative functionality. The reported *Nothofagus* transcriptome sequences represent a unique resource for genetic studies and provide a tool to discover genes of interest and genetic markers that will greatly aid questions involving evolution, ecology, and conservation using genetic and genomic approaches in the genus.

## Background

The Nothofagaceae family contains only the genus *Nothofagus*, and comprises 36 recognized species, 26 of which occur in Australia and the remaining 10 in South America [1]. *Nothofagus* in Argentina is represented by only six endemic species, distributed on the foothills of the Andes and surrounding valleys, beginning with its appearance at 36° in the province of Neuquen, and extending to 55°S, in the province of Tierra del Fuego [2].

Among these species, *N. obliqua*, *N. nervosa* and *N. pumilio*, occupy a relatively precise range within an altitudinal gradient spanning from 600 m over the sea level up to 1800 m. Along this gradient each species withstand different environmental conditions, especially extremely cold temperatures at the higher altitudes. Individual trees living in this environmental gradient, exhibit adaptive features for adverse conditions such as drought and extreme temperatures, traits that may prove value for adapting to future climate changes in the context of global climate change.

*N. nervosa (Phil*.) *Dim.et Mil*[3] (= *N. alpina (Poepp. &Endl.) Oerst)* commonly known as “raulí”, is one of the most important species of Patagonian Temperate Forests due to its wood quality and its relatively fast growth [4]. In Argentina it covers a reduced area, only 79,636 hectares in a narrow fringe of about 120 km in length and about 40 km in maximum width [5,6]. This deciduous species suffered a great overexploitation in the past due to its high wood quality, making necessary to implement conservation policies and management programs [7].

The distribution of adaptive genetic variation is an importance issue in forest species, both native and domesticated, serving as a basis for natural resource management and conservation genetics [8]. The characterization of genetic diversity is also important in order to determine its relation with phenotypic variation [9]. Massive sequencing techniques are among the new strategies used in functional genomics for gene discovery and molecular markers development in non-model organisms or in those species whose genomes have not been completely sequenced. It provides a fast and effective way to get new genetic information of an organism and allows a rapid access to a collection of expressed sequences (transcriptome).

To date, model forest tree species belonging to *Eucalyptus* genus [10-12], *Pinus*, *Picea* and *Populus*[13-17] have comprehensive transcriptome information.

The Fagaceae family (represented by the genus *Quercus*, *Castanea* and *Fagus*) also holds a large number of sequenced transcripts with approximately 2.5 millions of ESTs deposited in databases (Fagaceae Genomics Web: http://www.fagaceae.org/). At present, new sequencing technologies offer the possibility to obtain gene catalogs for non-model organism which is an opportunity for forest tree transcriptome characterization, discovery of alternative metabolic strategies and functional molecular markers [9].

One of the advantages of transcriptome pyrosequencing is in terms of sequence reliability. Each region of the cDNA is read several times in both strands compared to one sequence/one strand reading of conventional ESTs.

In this study we characterized leaf *N. nervosa* transcriptome by pyrosequencing and analyzed the resulting sequence data. Moreover, the functional annotation of the unigenes, allowed us to have a global but throughout picture of leaf functional gene expression, as well as to deduce the metabolic pathway represented in this dataset.

This information will significantly contribute to the development of *Nothofagus* functional genomics, genetics and population-based genome studies. In addition, the rather limited set of molecular markers available until now: 14 microsatellites isolated from *N. cunnighamii*[18], 11 developed in six species of South American *Nothofagus*[19], five in *N. nervosa*[20], and nine microsatellite loci from *N. pumilio*[21], will be substantially increased with thousands of new markers, both from neutral and functional sequences. The quality of the sequence information here reported was confirmed by the successful PCR amplification of molecular markers using oligonucleotide primers designed with the deduced sequences.

## Results and discussion

### Transcriptome sequencing and assembly

Pyrosequencing of cDNA on a 454 GS FLX Titanium (Roche) generated a total of 146,267 raw reads, with an average length of 408 bp. After filtering for adaptors, primer and low-quality sequences, 5,588 reads were removed resulting in 140,679 high quality reads corresponding to 96% of the first raw sequences, representing approximately 60 Mbp. Raw data (>200 bp) were deposited in NCBI Sequence Read Archive (SRA) under the accession number SRA049632.2.

**Figure 1:**
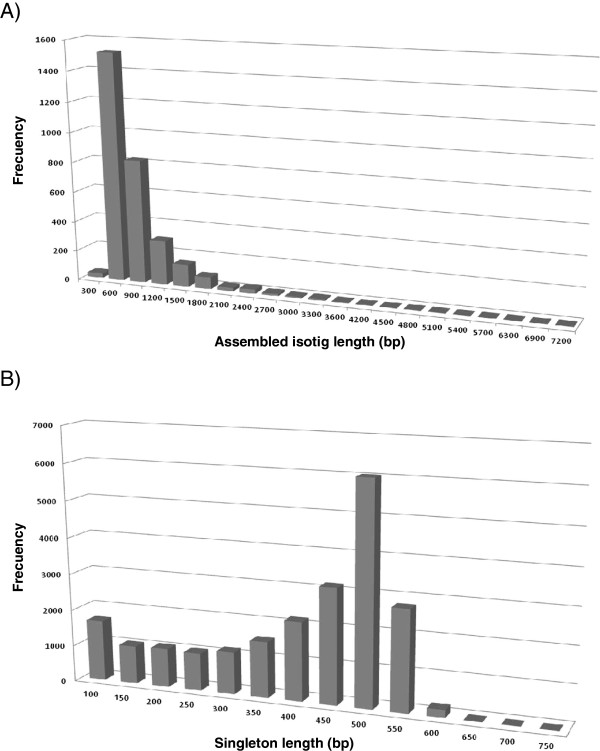
**Frequency distribution of isotigs (A) and singletons (B) sequences length.** The histograms represent the number of isotig and singletons sequences in relation to its length.

By using Newbler Software v. 2.5 (Roche, IN, USA); a total of 111,814 sequences were *de novo* assembled into 3,394 contiguous sequences (contigs). Overlapping contigs were assembled into 3,005 isotigs (equivalent to unique RNA transcripts). In addition, isotigs originating from the same contig-graph were grouped into 2,722 isogroups (equivalent to genomic locus) by Newbler, potentially reflecting multiple splice variants. About 28,861 reads not assembled into isotigs were clustered using CD-HIT-454 algorithm to eliminate artificial duplicates leaving 21,881 singletons, summing up a total of 24,886 non-redundant sequences or unigenes (Table 1). All unigene sequences (isotigs and singletons >200 bp) were deposited to the Transcriptome Shotgun Assembly (TSA) database, accession numbers JT763459-JT784547. Isotig length ranged from 66 bp to 7,093 bp, with an overall average length of 765 ± 537 bp (Figure 1A). More than 83% of the isotigs were 66 to 1,000 bp long and 50% of the assembled bases were incorporated into isotigs greater than 589 bp. The average length of *N. nervosa* isotigs (765 bp) was larger than those assembled in other non model organisms (e.g.197 bp [22], 440 bp [23], 500 bp [24]; 535 bp, [25]), and similar to the average isotig length described in *Bituminaria bituminosa* (707 bp [26]).

**Table 1:** ***N. nervosa *transcriptome annotation summary**

	**Number of sequences**		
	**Isotigs (3,005)**	**Singletons (21,881)**	**Combined set (24,886)**
**Viridiplantae-NR**			
Sequences with positive BLAST matches	2,762 (92%)	12,735 (58%)	15,497 (62%)
Sequences annotated with Gene Ontology (GO) terms	2,238 (74%)	9,596 (44%)	11,834 (47%)
Sequences without detectable BLAST matches	243 (8%)	9,146 (42%)	9,389 (38%)
Sequences assigned to know Enzyme Commission category	931 (31%)	1,424 (6%)	2,355 (9%)
**Fagaceae**			
Sequences with positive BLAST matches	2,923 (97%)	17,515 (80%)	20,438 (82%)
Sequences without detectable BLAST matches	82 (3%)	4,365 (20%)	4,447 (18%)
Sequences annotated with Gene Ontology (GO) terms (“novel genes”)	12 (0.4%)	490 (2%)	502 (2%)

Numbers and percentages of 454 sequences in the assembled isotigs, singletons and unigenes with significant matches against NCBI NR proteins Viridiplantae filtered database and Fagaceae unigenes.

The coverage depth for isotigs ranged from 2 to 19, with an average of 9 contigs assembled into each isotig, which is larger than the averages obtained in other 454 transcriptome analyses (mean = 2.1, [24,25]).

The length distribution of the 21,881 singletons ranged from 50 to 711 bp with an overall average length of 369.6 bp (Figure 1B). The length of 86% of the singletons was shorter than 500 bp.

### Functional annotation

All unique sequences were subjected to BLASTX similarity search against the NR protein database (National Center for Biotechnology Information, NCBI), with a Viridiplantae filter, to assign a putative function [27].

Under an E-value threshold of <10 ^-10^, a total of 2,762 isotigs (92% of total isotigs) and 12,735 singletons sequences (58% of total singletons) had significant BLASTX matches (Table 1). The frequency of annotated isotigs was significantly higher than the values previously reported for *de novo* transcriptome assemblies of eukaryotes that range from 20 to 40% [22-25].

In total, 15,497 unique sequences had at least one hit, while the remaining sequences 9,389 (38%) exhibited less significant matches (e-value > 10^−10^) but still informative for identifying putative biological functions in future studies in this species. We also performed a BLASTX against the NCBI - NR protein database to retrieve sequences that did not show BLAST hits against Viridiplantae NCBI, which summed up some few new hits (81), but not adding any other valuable annotations.

**Figure 2:**
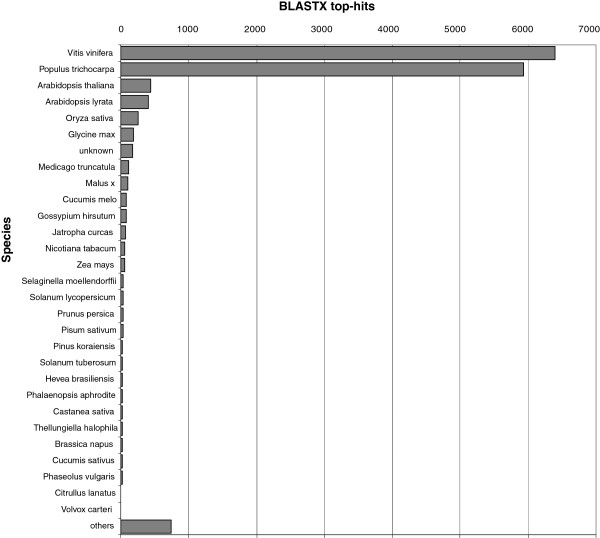
**Top-hit species distribution of BLASTX matches of *N. Nervosa *unigenes.** Proportion of *N. nervosa* unigenes (isotigs + singletons) with similarity to sequences from NCBI NR protein database (Viridiplantae and whole database).

The majority of matched sequences exhibited high similarity to *Vitis vinífera* (41%), and *Populus trichocarpa* (38%) sequences. The top-hit species distribution of BLAST matches is shown in Figure 2.

Annotation and mapping routines were run with BLAST2GO platform [28]. Sequences with a positive BLAST match were annotated using Gene Ontology terms (GO) and Enzyme Commission categories (i.e. EC numbers). Thus, GO terms were assigned to 2,238 isotigs (74%) and 9,596 singletons (44%) totalizing 11,834 GO terms (Table 1).

**Figure 3:**
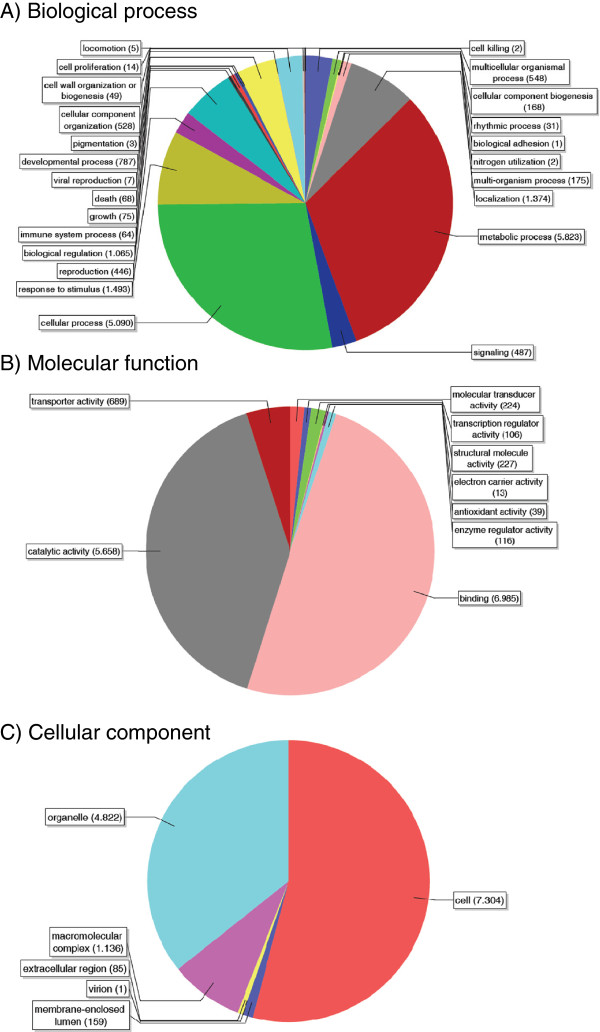
**Gene Ontology (GO) assignment in level 2 of 11,834** ***N. nervosa *unigenes.** The total numbers of unigenes annotated for each main category are 7,926 for “Biological Process” (**A**), 8,229 for “Molecular Function” (**B**), and 9,206 for “Cellular Component” (**C**).

Of the 11,834 GO annotated isotigs and singletons sequences, most were assigned to “Biological Processes” (7,926 terms), “Molecular functions” (8,229 terms) and “Cellular Components” (9,206 terms), (Figure 3).

BLAST2GO analysis at process level 2, showed that among 21 different biological processes most of the transcripts belonged either to “Metabolic Processes” (5,823), to “Cellular Processes” (5,090) and to “Response to Stimuli” (1,493), of which 756 were putative stress-response genes (Figure 3A).

Likewise, the molecular function category subdivided annotated sequences into binding (6,985), catalytic activity (5,658) and transporters (689) as the most represented (Figure 3B).

A detailed BLAST2GO analysis (level 2) at the cellular component category, sorted all transcripts from *N. nervosa* into 5 groups being the most representative: cell (7,304), organelle (4,822) and macromolecular complex component (1,136) (Figure 3C).

In order to more precisely compare the similarity of *N. nervosa* genes with those of the Fagaceae family (from Fagaceae Genomics Web [ http://www.fagaceae.org/]), *N. nervosa* unigenes were subjected to BLAT (dnax) search against 2,407,823 contigs and singletons from American Beech (*Fagus grandiflora*), American Chestnut (*Castanea dentate)*, Chinese Chestnut (*Castanea mollisima*) and oak species (*Quercus rubra* and *Q. alba*). Eighty-two percent of the *N. nervosa* expressed sequences exhibited high similarity to Fagaceae genes. A total of 4,447 (18%) sequences did not show matches against Fagaceae sequences, from which there were 82 isotigs and 4,365 singletons. Among them, 12 isotigs and 490 singletons had distinctive GO annotation, which could be considered as novel genes for this large group of tree species (Table 1). Most interestingly, from these transcripts 21 were found to be potentially new genes for stress response (data not shown).

Of the 11,834 sequences annotated with GO terms, 2,355 were assigned with EC numbers (931 isotigs and 1,424 singletons) (Table 1).

**Figure 4:**
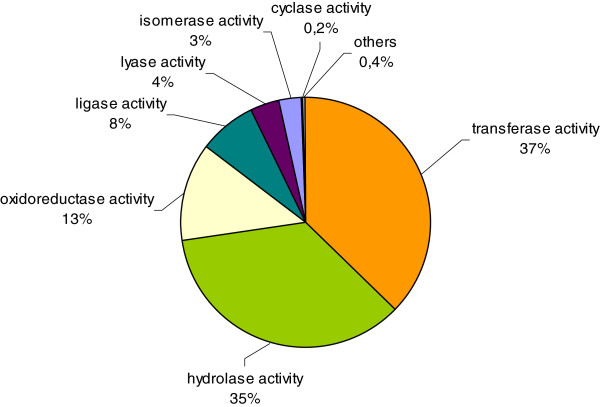
**Catalytic activity distribution in annotated *N. nervosa *unigenes.**

The most represented enzymes in all sequences are shown in Figure 4: transferase activity (37%), hydrolase activity (35%) and oxidoreductase activity (13%) were the most abundant.

To further enhance the annotation of *N. nervosa* transcriptome dataset, the 11,834 genes with GO terms were mapped to KEGG using KEGG automatic annotation server (KAAS) [29]. The identified 58 metabolic pathways include: purine metabolism (411), thiamine metabolism (405), T cell receptor signalling pathway (115), biosynthesis of secondary metabolites (58), and microbial metabolism in diverse environments (37) (see Additional file 1).

We detected as much as 861 chloroplast (cp) sequences (150 in isotigs and 711 in singletons), corresponding to a quite high rate (7%), but this value was within the 2 to 10% found in cDNA libraries from all tissue types, as reported in a study conducted in oak [30].

The number of annotated isotigs in this study was comparatively larger than that obtained in other similar studies [22-25]. These results could be associated with the high quality and small number of assembled isotigs, which potentially corresponds to highly expressed genes. Also the use of specific plant protein sequences and close related Fagaceae database possibly increased the BLAST hits. The first assumption comprises technical issues such as a high percentage of isotigs that was greater than ~600 bp length and with good coverage depth. Moreover, the small number of isotigs would be detecting the most represented and known expressed genes, as it was also shown in the analyses of *B. bituminosa* leaf transcriptome (89.1% annotated contigs) [26]. Proportions of best hits in major GO category were generally similar to those found in this species, for example, binding 48% and catalytic activity 37% in the *N. nervosa* transcriptome survey versus 37% and 37% respectively for the same categories in *B. bituminosa.*

The second statement relies on the annotation approach based on the search against the Viridiplantae protein database. This strategy allows to more likely finding BLAST hits above the cut off value. In addition, a higher percentage of reliable annotated isotigs was found when the searched was carried out against the Fagaceae protein sequence dataset (Table 1). The favorable effect of using specific databases for annotation was also reported for other authors [31-33].

Besides, the lower percentage of singletons that were annotated was likely due to the high frequency of short length sequences, also reported in recent studies [24,34]*.* Fifty percent of non-annotated singletons were shorter than 370 bp (data not shown), whereas the 50% in annotated singletons were longer than 454 bp. Similar results were obtained in *Pinus contorta* where only 5% of contigs and singletons had BLAST matches when the length of the sequences was less than 250 bp [24]. Nonetheless, many singletons were good quality reads and matched to proteins in BLAST searches representing together with the isotigs, a great source of information.

Summarizing, the frequency of annotated isotigs and singletons was significantly higher than previously reported for new generation sequencing *de novo* transcriptome assemblies of trees like *Pinus contorta*[24], or two oaks species, *Quercus petraea* and *Q. robur*[30], even though the high stringency of BLASTX analysis.

If we assume that the average number of genes encoded in a plant nuclear genome is about 30 thousands (as estimated from seven completely sequenced genomes) [34], our annotated dataset likely represents a half of the *N. nervosa* genes catalogue.

In order to test the presence of expressed repetitive sequences, BLASTN (e-value cut off ≤ 10e^-50^) searches were performed against all Viridiplantae Repbase (reference database of eukaryotic repetitive DNA). A total of 374 repetitive DNA sequences were found (57 in isotigs and 317 in singletons). From all the rRNA sequences, 255 corresponded to small subunit rRNA (SSUrRNA), 102 to large subunit rRNA (LSUrRNA) and 17 to transposable elements. Similar numbers of retrotransposon were observed in other plant species (e.g. 15 in *Populus tremula* and *Pinus pinaster*) [24]. However, in *Fagopyrum esculentum* and *Pinus contorta* much more transcribed retrotransposable elements were found in the different tissues sampled [24,34].

### *In silico* mining of single sequence repeats (SSRs)

Using the SSR webserver from the Genome Database for Rosaceae (GDR), we identified and characterized several SSRs (microsatellites) motives as potential molecular markers in the *Nothofagus* unigene collection.

The criteria used for SSR selection based on the minimum number of repeats was as follows: five for dinucleotide, four for trinucleotide, three for tetranucleotide and three for penta and hexanucleotide motives. These settings resulted in the identification of 3,821 putative SSRs within 24,886 unigenes i.e. SSR frequency of 15% considering multiple occurrences in a same unigene element. This was similar than that reported in oak 19% by Durand [35] and somewhat lower than 24%, estimated by Ueno [30]. A total of 3,048 (12%) unigenes contained at least one SSR, and 2,517 SSRs (66%) had sufficient flanking sequences to allow the design of appropriate unique primers. Information on the unigene identification (ID), marker ID, repeat motive, repeat length, primer sequences, positions of forward and reverse primers, and expected fragment length are included in Additional file 2.

### Characterization of microsatellite motives

**Figure 5:**
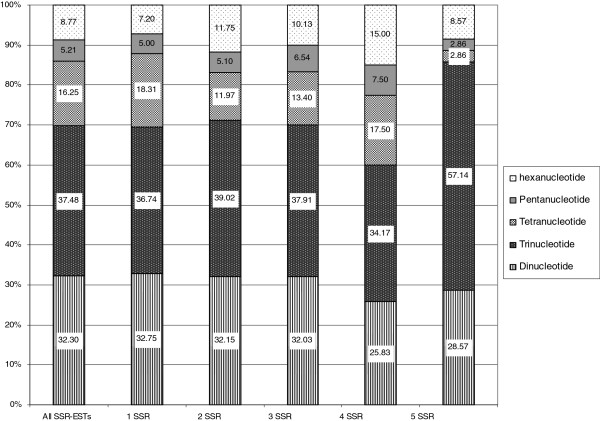
**Frequencies of SSR in *Nothofagus nervosa *unigenes.** Frequencies of di- tri- tetra- and penta-nucleotide SSRs in unigenes containing one to five SSRs.

As expected, the most frequent type of microsatellite corresponded to trimeric (37.4%) and dimeric motives (32.3%), being tetra-, penta- and hexanucleotide repeats present at much lower frequencies (16.3%, 5.2% and 8.8% respectively, Figure 5). Similar results were found in oak [30] (36.6% for trimeric and 36.2%, for dimeric motives) with the minimum repeat number of five and four for di- and tri-microsatellites, respectively.

SSR motif combinations can be grouped into unique classes based on DNA base complementarities. For example, dinucleotides were grouped into the following four unique classes: AT/TA; AG/GA/CT/TC; AC/CA/TG/GT and GC/CG. Thus, the numbers of unique classes possible for di-, tri- and tetra-nucleotide repeats are 4, 10, and 33, respectively [36,37]. The AG/CT group was the predominant class (56.2%) of the dinucleotide repeats, whereas AT (29.2%), AC (14.5%) and CG (0.1%) groups were less represented. The frequency of AG was similar to the highest value reported by Kumpatla [38] (14.6%–54.5% of the total SSRs observed in 55 dicotyledonous species) but lower than that found in Oak (70.5%) [30] and eucalypts (91%) [39].

The most frequent trimeric SSR motives were the AAG/CTT (27.8%), ATG/CAT (15.2), AGC/GCT (12.6%) and AGG/CCT (11.6%), similar to the first category found in oak (26.8%) [30]. Within tetrameric motives, AAAT repeat was found to be the most abundant (32.9%), followed by AAAG (22.7%) and AACA (11.6%).

The topography of SSR distribution was analyzed for SSR presence within UTRs and coding sequence regions. About 45% of the SSR sequences were inside ORF sequences. Most trinucleotide repeats were found in ORFs (52%), while dinucleotides were more frequent in the UTRs (40%), similar to those reported in oak [30] and pines [40]. It is expected that tri- and hexanucleotide repeats would occur more frequently than other motifs in coding sequences. Such dominance of triplets over other repeats in coding regions may be explained on the basis of the selective disadvantage of non-trimeric SSR variants in coding regions, possibly causing frame-shift mutations [41].

### Validation of the predicted microsatellite markers

Seventy three microsatellites were selected according to their sequence length, GC content and functional annotation related to abiotic stress category.

From these, 57% were located in coding regions. The 73 loci were tested for successful PCR amplification in six individuals*.* All of them were effectively amplified validating the quality of the assembly and the utility of the SSRs produced. A similar research carried using Illumina sequencing technology in sesame showed that about 90% primer pairs successfully amplified DNA fragments [42]. On the other side, the rate of SSR validation was lower (64.9%) when the marker mining was done using EST produced by Sanger technology [39] possibly because of low-quality EST sequences, and/or primer sequences derived from chimerical cDNA clones.

About 20% (14 SSR) of the tested *Nothofagus* SSRs were polymorphic and showed at least one individual that differed in allelic composition.

This relative low percentage of polymorphic loci could be explained because of the small sample size tested (six seedlings), in contrast to the 46% found in *E. globulus*[39] evaluated in 8 samples, and the 80% found in sesame [42] essayed in 24 samples.

Nine of the polymorphic SSR found in this work were located within predicted ORF and seven had repeat motives multiple of three (Table 2), according to their presence in coding regions [41].

**Table 2:** **Polymorphic SSRs primer pairs derived from *N. nervosa *unigenes**

**ID name**	**Locus**	**Repeat motif**	**ORF**	**Forward and Reverse Primers**	**Amplicon length observed**	**BLASTX, seq description**	**Seq Lenght (bp)**	**Sim mean (%)**	**GO terms related to response to stress**
isotig00192	INTANOT1	(tct)5	Y	F: CCAGATGGGTTTTTGCTTGT	148	heat shock protein 81-1	2309	97.2	response to stimulus
				R: GACGATGAAGACGATGAGC					
isotig00230	INTANOT2	(tcg)5	N	F: TTTCCAAACGGTTCCAGAAG	120	af367280_1at3g56860 t8m16_190	1229	76.6	response to stress
				R:AACGGAGAAGGATGTTTCCA					
isotig00551	INTANOT3	(tcattt)3	Y	F: CCGATGTGATCGATAGGCTT	204	ac005850_9highly simlilar to mlo proteins	1759	77.5	defense response to fungus
				R: CATGTCCCCAGTTCACCTCT					
isotig00597	INTANOT4	(ta)6	N	F:AAAACACCACCAAACCCAAA	197	dnaj heat shock n-terminal domain-containing protein	1516	78.3	response to stimulus
				R: CTTTGCCACGGCAACTAAAT					
isotig01207	INTANOT5	(tct)7	N	F: CTCGAAGACGCTACCAGACC	280	af214107_1 -like protein	748	79.3	response to stimulus
				R: TCCTGGGTTTTGCATATTGG					
isotig01232	INTANOT6	(atc)4	Y	F: CGTTTCCCTTTAGCTGATGC	173	aldh6b2 3-chloroallyl aldehyde dehydrogenase methylmalonate-semialdehyde dehydrogenase oxidoreductase	741	96.8	response to stress
				R:GCTGAGTTAGCAATGGAGGC					
GR7D2IN01BK031	INTANOT7	(ag)5	N	F: GACGACATCGTTCCGAGTTT	241	f-box family protein	536	75.4	response to heat
				R: GTTAATCCCTCTCTCCTCAT					
GR7D2IN01CGQUT	INTANOT8	(ccgaaa)3	Y	F: CTCCCTCAAACACCTCCAAA	236	mitogen-activated protein kinase kinase	518	90.5	response to osmotic stress
				R: ATTCAAGTGGGTCTTGCCTG					
GR7D2IN01EMGE0	INTANOT9	(ct)8	N	F: CCGGCTACCTGTTTGTTTTA	155	at1g78870 f9k20_8	507	100.0	response to metal ion
				R: TTCCTTGATGATTCTTCGGG					
GR7D2IN02FPPC7	INTANOT10	(ggt)6	Y	F: AAAATTGCTGTTGAGGGTGG	117	af361609_1at1g27760 t22c5_5	529	87.9	response to osmotic stress
				R: CCTGAATCACCAGACCGAC					
GR7D2IN02GFAUT	INTANOT11	(gaa)4	Y	F: ATCCCCAATCTTTCCCAATC	115	salt overly sensitive 1	315	78.5	response to reactive oxygen species; response to osmotic stress
				R: AATTCTGTCCGCTTTGGCTA					
GR7D2IN02GR6NZ	INTANOT12	(at)5	Y	F: TCTTGTGGCAAGTGCTTGAG	285	win2_soltu ame: full = wound-induced protein win2 flags: precursor	472	94.0	defense response
				R: ACTATCCTCACCGTTGCCTG					
GR7D2IN02HOKOI	INTANOT13	(tc)5	Y	F: ATATCCTGGAAATGCTTGCG	124	exec1_arath ame: full = protein executer chloroplastic flags: precursor	469	71.7	response to reactive oxygen species
				R: TAAACGATCTTCGGAATGGC					
GR7D2IN02HWXOR	INTANOT14	(tgg)8	Y	F: AGGAGCTAAATGGGCGTAA	260	glycine-rich rna-binding protein	452	86.5	response to stress
				R: CACCACCACCACCAAAGAA					

Included are ID names, primer names, motive and number of repeats, position in ORF, sequence of forward and reverse primers (5′3′), amplicon length (bp), BLASTX similarity matches (Putative Function), Sequence length, Similarity Mean (%), GO terms related to stress response.

## Conclusions

The transcriptome database obtained and characterized here represents a major contribution for *N. nervosa* genomics and genetics. It will be useful for discovering genes of interest and genetic markers to investigate functional diversity in natural populations, and as well as conduct comparative genomics studies in southern beeches taking advantage of their remarkable ecophysiological differences. This work highlights the utility of transcriptome high performance sequencing as a fast and cost effective way for obtaining rapid information on the coding of genetic variation in *Nothofagus* genus. This study allowed us to: (i) obtain 146,267 transcript raw reads and 24,886 unigene sequences from *N. nervosa*, (ii) identify putative function in 15,497 unigenes for the genus that potentially represent 50% of *N. nervosa* transcriptome, (iii) identify 756 putative stress-response genes (21 non described in Fagaceae), (iv) discover 2,517 SSRs with designed primers and (v) detect 14 polymorphic SSR related to stress response.

## Methods

### RNA preparation and cDNA library synthesis

Total RNA was prepared by the method of Chang and collaborators [43] from leaves of one single seedling. One gram of fresh tissue was used, ground to a fine powder under liquid nitrogen. Then, after 2 extractions with chloroform, RNA was precipitated with LiCl_2_, extracted again with chloroform and finally precipitated with ethanol. The resultant RNA was resuspended in 50 μl of DEPC treated water. RNA was quantified using a Nanodrop 1,000 spectrophotometer and the quality was measured with a 2,100 Bioanalyzer (Agilent Technologies Inc.) Total RNA isolated was purified using the Poly (A) Purist kit (Ambion) and the quality assessed with a 2,100 Bioanalyzer (Agilent Technologies). cDNA was synthesized using cDNA Kit (Roche) and used to construct a shotgun library for pyrosequencing technology (Roche). *Nothofagus* cDNA library was subjected to a 1/3 of plate production run on the 454-GS-FLX sequencing instrument. 454 library and sequencing was conducted at INDEAR (Rosario Biotechnology Institute, Rosario, Argentina).

### Transcript assembly and analysis

After removing low quality sequences, filtering for adaptors and primers, curated raw 454 read sequences were assembled into contigs, isotigs and isogroups using Newbler Assembler software 2.5p1 (Roche, IN, USA). Reads identified like singletons (i.e., reads not assembled into isotigs) after assembly, were subjected to CD-HIT-454 clustering algorithm using a sequence identity cut-off of 90%, which eliminates redundant sequences or artificial duplicates.

BLASTX (e-value cut off ≤ 10e^-10^) searches were performed against Viridiplantae protein database first, then the sequences with no hits were used to perform a successive BLASTX against the NCBI *nr* protein database in order to make an assessment of the putative identities of the sequences. Also we performed a pairwise alignment using BLAT (dnax) against the Fagaceae family sequences to search expressed sequence exclusively for *N. nervosa*. Annotation and mapping routines were run with BLAST2GO, which assigns Gene Ontology (GO; http://www.geneontology.org) annotation, KEGG maps (Kyoto Encyclopedia of Genes and Genomes, KASS) and an enzyme classification number (EC number) using a combination of similarity searches and statistical analysis [29].

To search for chloroplast sequences we performed BLASTN and TBLASTX (BLASTN e^-50^, TBLASTX 10e^-10^) by similarity (with and without translation) to 109 chloroplasts (nt and aa) from chloroplast genome data base ( http://chloroplast.cbio.psu.edu/organism.cgi).

### SSR discovery

In order to identify SSRs for all possible combinations of dinucleotide, trinucleotide, tetranucleotide and pentanucleotide repeats the SSR webserver (GDR) was run ( http://www.rosaceae.org/bio/content?title=&url=/cgi-bin/gdr/gdr_ssr). The same tool used GETORF algorithm (EMBOSS Package) to selected the longest ORF as the putative coding region, and Primer 3 (v.0.4.0) [44] to design primer pairs.

The presence of expressed repetitive DNA was performed using the BLASTN (e-value cut off ≤10e^-10^) searches against all Viridiplantae Repbase and CENSOR [45], a software tool that screens query sequences against a reference collection of repeats, and “censors” (masks) homologous portions with masking symbols, as well as generating a report classifying all found repeats.

### SSR validation

For validation of SSR primers, total DNA was extracted from young leaves of six *N. nervosa* seedlings using the Dneasy Plant mini kit (Qiagen), following the manufacturer’s instructions.

Regular primers at small scale were synthesized (AlphaDNA, Montreal, CA, USA) and used for PCR amplification. PCR reactions consisted of 20 ng total, 0.25 μM of each primer, 3 mM MgCl_2_, 0.2 mM of each dNTP, 1X PCR buffer and 1 U Platinum Taq polymerase (Invitrogen). All polymerase chain reactions amplifications were performed with the following conditions: denaturation step of 2 min at 94°C, a regular touchdown PCR ranging from 60°C to 50°C (except INTANOT14 (annealing at 55°C)) with 28 cycles at the touchdown temperature of 50°C according to: 45 s at 92°C, 45 s at 50°C and 45 s at 72°C. The final extension step was of 10 min at 72°C. Samples were mixed with denaturing loading buffer, incubated for 5 min at 95°C, and separated on a 6% polyacrylamide gel. Amplification products were stained using the DNA silver staining procedure of Promega, USA, following the manufacturer’s instructions. Details of primers sequences, SSR location and amplicon sizes are described in Table 2.

## Competing interests

The authors declare that they have no competing interests.

## Authors’ contributions

SLT organized the research, provided funds, contributed to RNA extraction, data analysis and wrote manuscript. MR carried out all bioinformatics analysis and contributed to draft the manuscript. MFP contributed to RNA extraction and SSR validation. PF contributed to RNA extraction and manuscript revision. CVA contributed to analyses involving BLAST, SSR characterization and contributed to draft the manuscript. PM provided the biological material for transcriptome sequencing and manuscript revision. SG assisted the bioinformatics analysis. MMA contributed to write the project and manuscript revision. LAG conceived this study and contributed to conceptual planning of the research. HEH conceived this study, assisted in the interpretation of the results and helped to draft the manuscript. NBP participated in the design of the study, supervised the bioinformatic analysis and reviewed the manuscript. SNMP provided funding, was involved in research design, SSR data analysis and contributed to draft and revision of the manuscript. All authors approved the final manuscript.

## Supplementary Material

Additional file 1 **KEGG Pathway maps.** This table provides information on the enzymes putatively encoded by the RNA sequences, based on homology prediction and their associated pathways. This includes KEGG maps, enzyme names, and sequences ID. Click here for file

Additional file 2 ***In silico*SSRs derived from*Nothofagus*leaf transcriptome (24,886 unigenes).** The data describe the 3,821 SSR: Included are unigenes names, marker ID, Sequence Length (bp), SSR description (# SSRs per seq, repeat length, motif, # Repeats, SSR position (start, stop)), ORF definition (start, stop, SSR in ORF), primers description (sequence of forward and reverse primers), expected product size (bp), similarity matches, E value, similarity mean, #GO, GO terms, Enzymes codes. Click here for file

## References

[B1] Promis A Cruz G Reif A Gartner S *Nothofagus betuloides* (Mirb.) Oerst 1871 (Fagales: Nothofagaceae) Forests in southern Patagonia and Tierra del Fuego Anales Instituto Patagonia (Chile) 2008 36 1 53 68

[B2] Guerra PE Stella RA, Ottone JR Especies nativas o autóctonas de los Bosques subantárticos In Maderas y Bosques Argentinos. Volume 2 2009 2 Orientación Gráfica Editora, Buenos Aires 975 1009

[B3] Lennon JA Martin ES Steven RA Wingston DL *Nothofagus nervosa* (Phil.) Dim. et Mil. The correct name for raulí, a chilean southern beech (*N. procera*) Arboricul 1987 11 323 332 10.1080/03071375.1987.9756364

[B4] Marchelli P Gallo L Scholz F Ziegenhagen B Chloroplast DNA markers reveal a geographical divide across Argentinean southern beech Nothofagus nervosa(Phil.) Dim. et Mil. distribution area TheorAppl Genet 1998 97 642 646 10.1007/s001220050940

[B5] Donoso C Bosques templados de Chile y Argentina Variación, Estructura y Dinámica 1993 Editorial Universitaria, Santiago de Chile

[B6] Sabatier Y Azpilicueta MM Marchelli P González-Peñalba M Lozano L García L Martinez A Gallo L Umaña F Bran D Pastorino M Distribución natural de *Nothofagus alpina y Nothofagus obliqua* (Nothofagaceae) en Argentina. Dos especies de primera importancia forestal de los bosques templados Norpatagónicos Bol Soc Argent Bot 2011 46 131 138

[B7] Marchelli P Gallo L Annual and geographic variation in seed traits of Argentinean populations of southern beech Nothofagus nervosa (Phil.) Dim. et Mil Forest Ecol Manag 1999 121 239 250 10.1016/S0378-1127(99)00004-3

[B8] Geburek T Turok J Conservation and management of forest genetics resources in Europe 2005 Arbora Press, Zvolen

[B9] Neale DB Kremer A Forest tree genomics: growing resources and applications Nat Rev Genet 2011 12 111 122 21245829 10.1038/nrg2931

[B10] Keller G Marchal T SanClemente H Navarro M Ladouce N Wincker P Couloux A Teulières C Marque C Development and functional annotation of an 11,303-EST collection from *Eucalyptus* for studies of cold tolerance Tree Genet Genomes 2009 5 317 327 10.1007/s11295-008-0184-7

[B11] Novaes E Drost DR Farmerie WG Pappas GJ Jr Grattapaglia D, Sederoff R, Kirst M: High-throughput gene and SNP discovery in *Eucalyptus grandis*, an uncharacterized genome BMC Genomics 2008 9 312 10.1186/1471-2164-9-312 18590545 PMC2483731

[B12] Mizrachi E Hefer CA Ranik M Joubert F Myburg AA De novo assembled expressed gene catalog of a fast-growing *Eucalyptus* tree produced by Illumina mRNA-Seq BMC Genomics 2010 11 681 10.1186/1471-2164-11-681 21122097 PMC3053591

[B13] Allona I Quinn M Shoop E Swope K Cyr SS Carlis J Riedl J Retzel E Campbell MM Sederoff R Whetten RW Analysis of xylem formation in pine by cDNA sequencing Proc Natl Acad Sci USA 1998 95 9693 9698 10.1073/pnas.95.16.9693 9689143 PMC21401

[B14] Li XG Wu HX Dillon SK Southerton SG Generation and analysis of expressed sequence tags from six developing xylem libraries in *Pinus radiate* D Don. BMC Genomics 2009 10 41 10.1186/1471-2164-10-41 PMC2636829 19159482

[B15] Pavy N Paule C Parsons L Crow JA Morency MJ Cooke J Johnson JE Noumen E Guillet-Claude C Butterfield Y Barber S Yang G Liu J Stott J Kirkpatrick R Siddiqui A Holt R Marra M Seguin A Retzel E Bousquet J MacKay J Generation, annotation, analysis and database integration of 16,500 white spruce EST clusters BMC Genomics 2005 6 144 10.1186/1471-2164-6-144 16236172 PMC1277824

[B16] Nanjo T Futamura N Nishiguchi M Igasaki T Shinozaki K Shinohara K Characterization of full-length enriched expressed sequence tags of stress-treated poplar leaves Plant Cell Physiol 2004 45 1738 1748 10.1093/pcp/pci009 15653793

[B17] Unneberg P Stromberg M Lundeberg J Jansson S Sterky F Analysis of 70,000 EST sequences to study divergence between two closely related *Populus* species Tree Genet Genomes 2005 1 109 115 10.1007/s11295-005-0014-0

[B18] Jones RC Vaillancourt RE Jordan GJ Microsatellites for use in Nothofagus *cunninghamii* (Nothofagaceae) and related species Mol Ecol Notes 2004 4 1 14 16

[B19] Azpilicueta M Caron H Bodénès C Gallo L SSR markers for analyzing South American *Nothofagus* species Silvae Genet 2004 53 240 243

[B20] Marchelli P Caron H Azpilicueta M Gallo L A new set of highly polymorphic nuclear microsatellite markers for Nothofagus nervosa and related South American species Silvae Genet 2008 57 2 82 85

[B21] Soliani C Sebastiani F Marchelli P Gallo L Vendramin GG Development of novel genomic microsatellite markers in the southern beech Nothofagus pumilio (Poepp. et Endl.) Krasser Mol Ecol, Resources 2010 10 404 408

[B22] Vera JC Wheat CW Fescemyer HW Frilander MJ Crawford DL Hanski I Marden JH Rapid transcriptome characterization for a non model organism using 454 pyrosequencing Mol Ecology 2008 17 1636 1647 10.1111/j.1365-294X.2008.03666.x 18266620

[B23] Meyer E Aglyamova GV Wang S Buchanan-Carter J Abrego D Colbourne JK Willis BL Matz MV Sequencing and de novo analysis of a coral larval transcriptome using 454 GSFlx BMC Genomics 2009 10 219 1 18 19435504 10.1186/1471-2164-10-219 PMC2689275

[B24] Parchman TL Geist KS Grahnen JA Benkman CW Buerkle CA Transcriptome sequencing in an ecologically important tree species: assembly, annotation, and marker discovery BMC Genomics 2010 11 180 10.1186/1471-2164-11-180 20233449 PMC2851599

[B25] Rismani-Yazdi H Haznedaroglu BZ Bibby K Peccia J Transcriptome sequencing and annotation of the microalgae Dunaliella tertiolecta: Pathway description and gene discovery for production of next-generation biofuels BMC Genomics 2011 12 148 10.1186/1471-2164-12-148 21401935 PMC3061936

[B26] Pazos-Navarro MD Correal E Hanson H Teakle N Real D Nelson MN Next generation DNA sequencing technology delivers valuable genetic markers for the genomic orphan legume species Bituminaria bituminosa. BMC Genet 2011 12 104 10.1186/1471-2156-12-104 PMC3265443 22171578

[B27] Gish W States DJ Identification of protein coding regions by database similarity search Nat Genet 1993 3 3 266 272 10.1038/ng0393-266 8485583

[B28] Conesa A Götz S García-Gómez JM Terol J Talón M Robles M BLAST2GO: a universal tool for annotation, visualization and analysis in functional genomics research Bioinformatics 2005 21 3674 3676 10.1093/bioinformatics/bti610 16081474

[B29] Moriya Y Itoh M Okuda S Yoshizawa AC Kanehisa M KAAS: an automatic genome annotation and pathway reconstruction server Nucleic Acids Res 2007 35 182 185 10.1093/nar/gkm321 PMC1933193 17526522

[B30] Ueno S Le Provost G Léger V Klopp C Noirot C Frigerio JM Salin F Salse J Abrouk M Murat F Brendel O Derory J Abadie P Léger P Cabane C Barré A de Daruvar A Couloux A Wincker P Reviron MP Kremer A Plomion C Bioinformatic analysis of ESTs collected by Sanger and pyrosequencing methods for a keystone forest tree species: oak BMC Genomics 2010 11 650 10.1186/1471-2164-11-650 21092232 PMC3017864

[B31] Leroy P Guilhot N Sakai H Bernard A Choulet F Theil S Reboux S Amano N Flutre T Pelegrin C Ohyanagi H Seidel M Giacomoni F Reichstadt M Alaux M Gicquello E Legeai F Cerutti L Numa H Tanaka T Mayer K Itoh T Quesneville H Feuillet C TriAnnot: a versatile and high performance pipeline for the automated annotation of plant genomes Front Plant Sci 2012 3 5 22645565 10.3389/fpls.2012.00005 PMC3355818

[B32] Barakat A DiLoreto DS Zhang Y Smith C Baier K Powell WA Wheeler N Sederoff R Carlson JE Comparison of the transcriptomes of American chestnut (*Castanea dentata*) and Chinese chestnut (Castanea mollissima) in response to the chestnut blight infection BMC Plant Biology 2009 9 51 10.1186/1471-2229-9-51 19426529 PMC2688492

[B33] Faria-Campos AC Campos SV Prosdocimi F Franco GC Franco GR Ortega JM Efficient secondary database driven annotation using model organism sequences In Silico Biol 2006 6 5 363 372 17274765

[B34] Logacheva MD Kasianov AS Vinogradov DV Samigullin TH Gelfand MS Makeev VJ Penin AA De novo sequencing and characterization of floral transcriptome in two species of buckwheat (*Fagopyrum*) BMC Genomics 2011 12 30 10.1186/1471-2164-12-30 21232141 PMC3027159

[B35] Durand J Bodénès C Chancerel E Frigerio JM Vendramin G Sebastiani F Buonamici A Gailing O Koelewijn HP Villani F Mattioni C Cherubini M Goicoechea P Herrán A Ikaran Z Cabané C Alberto F Dumoulin PY Guichoux E de Daruvar A Kremer A Plomion C A fast and cost-effective approach to develop and map EST-SSR markers: oak as a case study BMC Genomics 2010 11 570 10.1186/1471-2164-11-570 20950475 PMC3091719

[B36] Jurka J Pethiyagoda C Simple repetitive DNA sequences from primates: compilation and analysis J Mol Evol 1995 40 2 120 126 10.1007/BF00167107 7699718

[B37] Katti MV Ranjekar PK Gupta VS Differential distribution of simple sequence repeats in eukaryotic genome sequences Mol Biol Evol 2001 18 7 1161 1167 10.1093/oxfordjournals.molbev.a003903 11420357

[B38] Kumpatla SP Mukhopadhyay S Mining and survey of simple sequence repeats in expressed sequence tags of dicotyledonous species Genome 2005 48 985 998 10.1139/g05-060 16391668

[B39] Acuña CV Fernandez P Villalba PV García MN Hopp HE Marcucci Poltri SN: Discovery, validation, and in silico functional characterization of EST-SSR markers in *Eucalyptus globulus* Tree Genet Genomes 2012 8 289 301 10.1007/s11295-011-0440-0

[B40] Chagné D Chaumeil P Ramboer A Collada C Guevara A Cervera MT Vendramin GG Garcia V Frigerio JM Echt C Richardson T Plomion C Cross-species transferability and mapping of genomic and cDNA SSRs in pines Theor Appl Genet 2004 109 1204 1214 10.1007/s00122-004-1683-z 15448894

[B41] Metzgar D Bytof J Wills C Selection against frameshift mutations limits microsatellite expansion in coding DNA Genome Res 2000 10 1 72 80 10645952 PMC310501

[B42] Wei W Qi Xi Wang L Zhang Y Hua W Li D Lv H Zhang X Characterization of the sesame (*Sesamum indicum* L.) global transcriptome using Illumina paired-end sequencing and development of EST-SSR markers BMC Genomics 2011 12 451 10.1186/1471-2164-12-451 21929789 PMC3184296

[B43] Chang S Puryear J Cairney J A simple and efficient method for isolating RNA from pines trees Plant Mol Biol Rep 1993 11 2 113 116 10.1007/BF02670468

[B44] Rozen S Skaletsky HJ Primer 3 on the WWW for general users and for biologist programmers Methods Mol Biol 2000 132 3 365 386 10547847 10.1385/1-59259-192-2:365

[B45] Kohany O Gentles AJ Hankus L Jurka J Annotation, submission and screening of repetitive elements in Repbase: RepbaseSubmitter and Censor BMC Bioinformatics 2006 7 474 10.1186/1471-2105-7-474 17064419 PMC1634758

